# The Matrix Matters: Beverage Carbonation Impacts the Timing of Caffeine Effects on Sustained Attention

**DOI:** 10.3390/nu14112305

**Published:** 2022-05-31

**Authors:** Evelina De Longis, Clara Lerond, Sarah E. Costello, Julie Hudry

**Affiliations:** Brain Health Department, Nestlé Institute of Health Sciences, Nestlé Research, Société des Produits Nestlé S.A., 1000 Lausanne, Switzerland; clara.lerond@rd.nestle.com (C.L.); sarah.costello@rd.nestle.com (S.E.C.); julie.hudry@rdls.nestle.com (J.H.)

**Keywords:** caffeine, sustained attention, carbon dioxide, refreshing, mental energy, fatigue

## Abstract

Both caffeine and the perception of refreshment delivered by cooling, tingling, and mouth-watering flavors have individually been shown to positively impact cognitive performance and mood, though presently there is limited evidence on their possible combined effects. This study explored the contribution of refreshing compounds in beverages, namely, carbon dioxide and citric acid, on the acute effects of caffeine on sustained attention and self-rated physical and mental energy. A randomized, controlled crossover trial was conducted by testing three products: a carbonated caffeinated beverage; a comparator caffeinated beverage; and a flavor-matched control beverage. Findings from 24 healthy adults revealed product-dependent variations in cognitive performance during a 60-min visual sustained-attention task, suggesting that the carbonated-caffeinated beverage led to faster, greater and more consistent levels of accuracy, compared to the control beverage. Specifically, significant differences were found between: (1) the carbonated-caffeinated beverage and the caffeinated beverage, and (2) between the caffeinated beverage and the control beverage for number of hits, reaction time and false alarm scores. Both caffeinated beverages led to higher physical and mental energy, and lower physical and mental fatigue 60-min post-consumption. These findings suggest beneficial effects on sustained attention through the combination of caffeine with refreshing compounds.

## 1. Introduction

It is well-established that caffeine consumption leads to an increase in attention and alertness [1,2,3]. Specifically, a large number of studies have shown caffeine to be effective in increasing and maintaining sustained attention, whilst also counteracting the effect of fatigue [4,5,6]. The effects of caffeine are most commonly assessed using standardized laboratory tasks, though studies have also shown positive associations with self-rated alertness and activation states [4,7].

Overall, the effects of caffeine on cognitive functions have proven to be beneficial through physiological effects mediated via changes in the cardiovascular, respiratory, and nervous systems [8]. Upon ingestion, caffeine is absorbed from the gastrointestinal tract with peak plasma concentrations reached 30 min after consumption [9]. The ability of caffeine to act as an adenosine antagonist is widely recognized as its primary mechanism of action, resulting in wake-promoting effects as well as in increased attention, and physical and mental energy [1,9,10,11].

Although the effects of caffeine on attention are well-acknowledged and despite several attempts at understanding the combined effects of caffeine with other ingredients on cognitive function, greater research is required to determine the role of the vehicle (i.e., the nature of the drink) with which caffeine is administered as this has been shown to affect the impact of caffeine on mood and cognition [12,13].

Among the factors that proved to have beneficial and rapid effects on attention, mood and mental energy, is the refreshing perception resulting from sensory properties such as cooling, tingling and mouth-watering flavors [14,15,16,17]. For instance, in the study conducted by Labbe et al. [14], an optimized water containing a cooling agent and citric acid was found to induce improvements in subjective alertness, performance of attention and cortical activation in the alpha and beta powers involved in cognitive processing. Interestingly, such improvements were observed 15 min post-consumption, showing a relatively fast efficacy of refreshing ingredients in improving cognitive performance [14]. Another refreshing compound that has been found to affect the time-course of energizing ingredients in beverages is carbon dioxide (CO_2_) [2]. CO_2_ is a colorless gas that, added as an ingredient to various beverages, such as carbonated soft drinks and sparkling waters, causes small bubbles to form, giving the product an effervescent quality. Two studies conducted by Smit et al. [2] suggested that carbonation is likely to promote longer lasting effects of caffeine on reaction times, as well as on feelings of energy. 

In the light of these results, CO_2_ and citric acid are good candidates to examine in relation to psychomotor performance, due to their potential acute effect on attention through trigeminal stimulations [18]. Indeed, while citric acid stimulates salivary glands in favor of perceived refreshment (i.e., a mouthwatering effect), CO_2_ in the mouth stimulates the trigeminal system via vanilloid TRPV1 and TRPA1 [18,19,20]. The trigeminal and gustatory pathways activate the noradrenergic system via vagal afferents as well as the sympathetic nervous system, possibly leading to immediate acute effects on attention [21,22,23,24]. Furthermore, neuro-imaging studies have shown that oral CO_2_ triggers the immediate activation of a wide network of brain regions mediating alertness and cognitive functions [25,26]. For example, other trigeminal stimulations, such as the cooling compound of mint (menthol, TRPM8 agonist), have been consistently associated with rapid and immediate stimulatory effects in humans, including reduced sleepiness [27], increased sustained attention [28,29,30], improved alertness [30] and enhanced physiological arousal (cortical excitability) [31,32,33]. Despite this evidence, it is surprising that only one study [2] has investigated the acute effect of a carbonated caffeinated product on mood and cognition. 

The purpose of this trial is to explore acute effects of caffeine in ingredient matrices with and without refreshing cues, on continuous performance during a sustained attention task and feelings of physical and mental energy. We hypothesized that a carbonated-caffeinated beverage, compared to a caffeinated beverage alone, would positively contribute to the time-course and maintenance of sustained attention.

## 2. Materials and Methods

### 2.1. Design

The trial followed a randomized, single-blind, cross-over design in 24 healthy adults with three arms: a test product (carbonated-caffeinated beverage, CCB), an energy comparator (caffeinated beverage, CB) and a flavor-matched control (control beverage). CCB and CB contained 82.2 mg of caffeine and 6.6 g of sugar in carbonated and still water, respectively. CCB also contained 0.6 g of citric acid. The control beverage was matched for sweetness perception using 1.1 g stevia and contained the same pomelo aroma. Each beverage had a total volume of 330 mL. The dose of caffeine was selected based on the EFSA [3] recommendations for physiological effects related to improvements in alertness and concentration. 

The research protocol was approved by the Human Ethics Review Committee of Vaud University Hospital Center in Lausanne, Switzerland (Swissethics). The voluntary trial participation was performed according to the guidelines of the Declaration of Helsinki 2000.

### 2.2. Participants

Twenty-four participants (*n* = 10 female) were recruited from the staff of Nestlé Research in Lausanne (Switzerland).Participant characteristics can be seen in Table 1. Participants gave written informed consent and received financial compensation for participating in the study. Medical screening for study inclusion was performed by a medical doctor and a research nurse. All participants were reported to be in good health, non-smokers, free of food allergies or intolerance, and with no condition or treatment that could affect functions of the autonomic nervous system, cognitive function, or perception. 

### 2.3. Procedure

**Figure 1:**
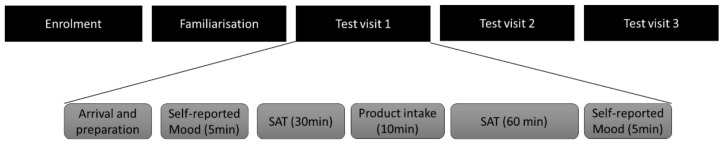
Overview of the study schedule.

Following a familiarization session, participants were required to attend three testing sessions, each separated by a washout period of at least 6 days (see Figure 1). The practice session aimed at familiarizing participants with the study protocol and experimental conditions to avoid any effects of practice and surprise related to the treatments or to the task duration during the first test session. To minimise distraction, all testing took place in a soundproof room. The visual selective-attention task was administered via a computer (via E-Prime software package, E-prime 1.1, Psychology Software Tools, Summit Software Company) and the behavioral responses of the participants were collected with a touch-sensitive response box. More details on the vigilance task are provided in the ‘*Measures*’ section.

EEG data and heart rate were also collected, but this data will not be presented in this paper. Participants received either the CCB, CB or control 30 min after completing 8 blocks of the SAT to induce initial fatigue [34,35]. They then continued with a 60 min SAT completing 16 blocks.

Participants were asked to refrain from drinking alcohol and any beverages containing caffeine (including coffee, tea, cola, or other soft drinks) overnight prior to each test session and to refrain from consuming food after 8 am the morning of the visit. 

### 2.4. Measures

- Sustained-Attention Task

Sustained attention was measured using a 90-min (30 min before product intake and 60 min after ingestion) continuous psychometric task of visual selective attention, widely used to investigate the impact of mental fatigue [36,37]. The task comprised of 24 4-min blocks of 160 trials. Each experimental block began with the presentation of a memory set of two letters (“target stimuli”) as well as a diagonal cue (“stimulus relevance”). Following this, a series of two letters were presented in rapid succession on the screen in diagonal positions with an interstimulus interval varying randomly between 1000 and 1500 ms. Participants were asked to respond as quickly and accurately as possible by pressing a touch-sensitive response box when the target stimulus was detected in the correct diagonal position. All four stimulus types (relevant target, relevant non-target, irrelevant target, and irrelevant non-target) appeared in a random order in 25% of the trials. 

Outcome measures for the sustained-attention task were accuracy (i.e., number of hits); mean response/reaction times for hit trials (i.e., relevant target detected as such) and mean error rates (expressed as a percentage of missed targets and of false positive responses) obtained for each experimental block following product intake.

b. Energy and fatigue

Energy and fatigue were assessed using the standardized “Mental and Physical State and Trait Energy and Fatigue” (MPS-TEF) scale [38], consisting of twelve 100 mm visual-analog scales items that measure four energy and fatigue states (score range 0–300). Three scales are summed for each of the following: Physical energy (energy, vigor, pep), Physical fatigue (fatigue, exhaustion, worn out), Mental energy (energy, vigor, pep) and Mental fatigue (fatigue, exhaustion, worn out). These dimensions were assessed at baseline and 60 min post-product consumption. 

### 2.5. Statistical Analysis

All statistical analyses were conducted using statistical software R (version 3.5.2) and SAS Life Sciences Analytics Framework. 

Per protocol evaluation data from participants was excluded in the following conditions: incomplete data (*n* = 1) and/or performance errors on the vigilance task with more than 2/24 consecutive blocks missed in each test session (*n* = 1); 22 participants were left in the final behavioral data analyses. 

To examine changes in *number of hits* during the 90 min task as a function of product and time, the post-treatment difference between products was assessed using a generalized mixed effect model with a logistic link function. To examine changes in *mean reaction times,* the post-treatment difference between products was assessed using a linear mixed model with a logistic link function. In both models, the following were entered as independent variables: product, baseline value, age, block and sequence, in addition to the two interaction terms: (1) block and product, and (2) sequence and treatment (to evaluate carry-over effect). The within-subject variability was accounted for by including participant ID as a random effect.

The *p* values were estimated via likelihood-ratio tests and the odds ratio (OR) statistics were calculated for interpretation of effect sizes.

The effect of the investigational products on feelings of energy and fatigue were analyzed using a mixed model with repeated measurements for each dimension (Physical energy, Physical fatigue, Mental energy, Mental fatigue). In these models, product, baseline (i.e., energy and fatigue ratings before product intake), age and sequence were entered as independent variables. As in previous analyses, a subject-specific random effect was specified to account for within-subject variability.

## 3. Results

### 3.1. Changes in Performance of Sustained Attention Task as a Function of Time-On-Task and Product

In line with our hypothesis, statistical analyses revealed a significant main effect of product on all SAT endpoints: number of hits and mean reaction times (*p* < 0.001). A significant effect of factor Block, indicating the effect of time, was found on number of hits and number of false alarms.

**Figure 2:**
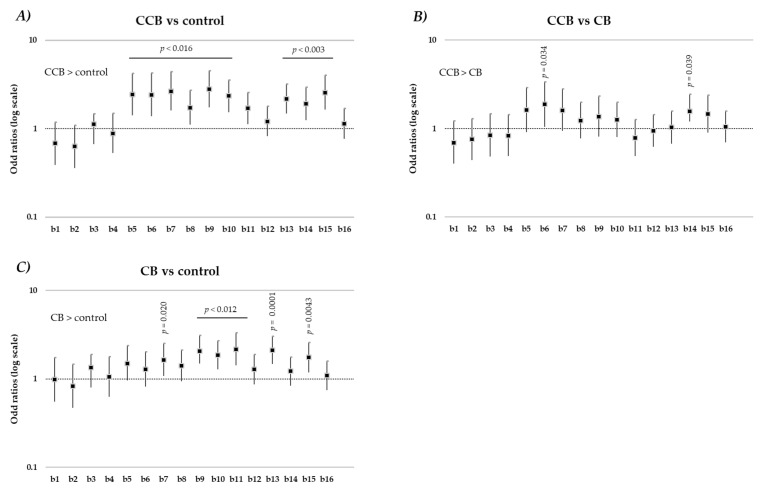
Odds ratios and 95% confidence intervals for product-effect differences on SAT accuracy for the 16 experimental blocks following product intake. Pairwise comparisons for accuracy are shown separately for carbonated caffeinated beverage and control beverage (**A**); carbonated caffeinated beverage and caffeinated beverage (**B**); caffeinated beverage and control beverage (**C**). CCB = carbonated caffeinated beverage; CB = caffeinated beverage; b = block.

Specifically, for number of hits, a significant difference was observed between (1) CCB and control beverage (OR = 1.6, C.I. 1.39, 1.84, *p* < 0.0001) and (2) CB and control (OR = 1.42, C.I. 1.25, 1.61, *p* < 0.0001). The interaction between product and block was statistically significant (*p* < 0.0001), suggesting that the type of product affected the trajectory of performance accuracy over time. Figure 2 shows product differences in number of hits over time as odd ratios: panel A shows the comparison between the CCB and the control beverage; panel B shows differences between CCB and CB; and panel C shows differences in accuracy between CB and control beverage. Compared to control, accuracy is higher following CCB and CB consumption. Of interest is that CCB led to earlier, more frequent differences in accuracy levels than control beverage. Significant differences were observed in 10 of the 16 blocks, and specifically from block 5 (around 15 min after intake) to block 11, and from block 13 to block 16 post-consumption (see Figure 2, panel A). Compared to CB, ingestion of CCB resulted in the greatest levels of accuracy in two blocks (i.e., block 6 and block 14; see Figure 2, panel B). Statistically significant differences were also observed between CB and control for 6 blocks: at block 7 (around 25 min after intake); from block 9 to 11; at block 13 and at block 15 (see Figure 2, panel C). Taken together, these results suggest that consumption of the CCB resulted in immediate and more frequent improvements in accuracy, compared to CB and control beverage. 

Similarly, for mean reaction times, pairwise comparisons showed a significant difference between (1) CCB and control (OR = −12.06, C.I. −16.64, −7.49, *p* < 0.0001) and (2) CB and control (OR = 16.66, C.I. −21.23, −12.09, *p* < 0.001). The interaction between product and block was not significant (*p* > 0.05). 

Finally, we found a significant difference in the number of false alarms only between CB and control (OR = 0.08, C.I. 0.67, 0.9, *p* < 0.001). The interaction between product and block was not significant (*p* > 0.05).

In all models, a product by age interaction was also tested, but it was not significant.

### 3.2. Effects of Product on Ratings of Energy and Fatigue

All four dimensions were normally distributed. Results from these analyses are presented in Table 2. As can be seen, we found a significant treatment effect on all dimensions. Specifically, in all models, a significant difference was observed between (1) CCB and control; and (2) CB and control. No significant differences were observed between CCB and CB. After the 90 min SAT, for CCB, we observed higher levels of physical and mental energy, as well as lower levels of mental and physical fatigue, compared to control. The lowest levels of mental and physical energy were observed in the control condition, which was also associated to high levels of physical and mental fatigue at the completion of the SAT.

## 4. Discussion

The purpose of this trial was to explore acute effects of caffeine in ingredient matrices with and without refreshing cues on continuous performance during a sustained-attention task and feelings of physical and mental energy. Following the results of previous studies [2,14] showing a quick impact of refreshing perception on mood and cognition, we hypothesized that CO_2_ and citric acid were likely to affect the time-course of the effects of caffeinated beverages on sustained attention. In support of this, results showed a significant interaction between product and blocks in predicting changes in accuracy during the SAT. By analyzing post-treatment differences in kinetics of performance in sustained attention, we found that the inclusion of refreshing compounds (i.e., CO_2_ and citric acid) led to faster and greater improvement in accuracy, compared to CB. Indeed, CCB was associated with an increased accuracy starting from about 15 min after product intake (i.e., from block 5, until block 11); the consumption of CB, on the other hand, led to an improvement in accuracy after 25–30 min post-ingestion (at block 7 and from blocks 9 to 11) in comparison to control. In addition, it was the consumption of CCB, rather than CB, that led to consistently greater levels of accuracy. Overall, the consumption of CCB led to faster, higher, and more consistent levels of accuracy. These findings are in-line with previous studies examining the impact of carbonation and refreshing perception on cognitive performance [2,14], suggesting that the sensory drivers of refreshing perception are likely to have fast effects on cognitive performance and mental energy. Administering caffeine in combination with CO_2_ can therefore lead to a more “immediate” and a more consistent effect on sustained attention, compared to caffeine alone, which generally reaches a peak plasma concentration at about 30 min post-intake. Seeing a quicker effect is likely to be driven by the sensory properties of the beverage and/or expectation effect [2,14]. The significant product by block interaction we observed in accuracy, but not in reaction time nor number of false alarms, is also consistent with the findings of Ploutz-Snyder et al. [39], who suggest carbonated beverages might be absorbed more slowly than when administered in a non-carbonated beverage. The authors found that the carbonation level in a drink accounted for 84% of the variation in gastric emptying time and is therefore the most important regulator of gastric emptying among the considered factors (carbonation, carbohydrates content, osmolality). This may suggest that the energizing ingredient (e.g., caffeine) contained in a carbonated beverage may be absorbed more slowly and thus result in longer-lasting effects on mood and cognition [2]. However, such a conclusion was not fully supported by the two studies conducted by Smit et al. [2]. In one study (Study 2), the authors found that carbonation promoted the energizing effect (i.e., feeling “awake”) of an energy drink beyond 80 min post-treatment testing times [2]. Nevertheless, in another study (Study 3), the authors [2] reported a decreased score on a rapid visual-information processing task at 45 min post-consumption of a carbonated energy drink, compared to a non-carbonated one. Moreover, while in Study 2 the authors observed a reduced immediate energizing effect of the carbonated beverage, in Study 3 carbonation resulted in immediate benefits on feelings of energy, most likely driven by a sensory effect [2]. These results may be due to confounding factors, or may suggest that the effects of carbonation are independent of those of other ingredients [2]. Our findings are in line with those of the study conducted by Ridout et al. [40], which offer further support for an immediate effect of CO_2_ on cognition, by suggesting that CO_2_ content in champagne may accelerate and enhance the rate of absorption of ethanol. The authors reported that champagne was associated with higher blood alcohol concentration and significantly slower reaction times in a divided attention task at 20- and 50-min post-consumption, compared to degassed champagne. This may, again, suggest absorption/gastric emptying times to be possible mechanisms through which carbonation can have an impact on the time-course of a beverages effect on mood and cognition [2,39,40]. Other mechanisms underlying the quick impact of refreshing oral stimulation on mood and cognition include trigeminal stimulation [18,25,30] and the resulting enhancement of brain oscillations, related to the activation of specific neural networks [14]. Alpha oscillations have indeed been identified as key components of attentional processes and alertness [41]. Consequently, such changes in brain activation induced by the sensory properties of refreshing perception seem to represent a physiological resource for the performance of sustained attention [14].

As for the main effect of product, in line with previous studies [1,4,5], our findings indicate significant product effects on accuracy, reaction time and number of false alarms, suggesting an improvement in sustained attention following caffeine ingestion. Overall, the accuracy was greater following ingestion of CCB compared to CB and the control beverage, and reaction times were faster following caffeine intake. Of interest is that accuracy and number of false alarms, but not reaction time, showed variation over time.

It is important to note that we did not observe any statistically significant difference in the SAT endpoints between CCB and CB. Indeed, we observed significant differences between CCB and control, and between CB and control. This result is not surprising, however, as it is consistent with the well-established literature that caffeine is the major driver of improvements in performance of attention [2].

Another key finding of this study is related to the product effect on self-rated energy and fatigue levels 60 min after consumption, after completing the SAT. As seen for the SAT results, we found statistically significant differences in energy and fatigue ratings between CCB and control and the CB and control. This finding suggests that caffeine was the main driver for higher feelings of physical and mental energy and lower levels of physical and mental fatigue. This result is consistent with previous research on the association between caffeine and self-rated mood [4,7], as well as with the idea that sensory perception is more likely to have a quick and immediate impact on subjective feelings due to its physiological benefits [14], which we could not capture at 60 min after product ingestion. It can be speculated that an effect of refreshing perception could have been captured at shorter post-ingestion assessments. 

Two strengths of this study are the well-controlled design and the inclusion of the initial 30-min cognitive task to induce mental fatigue before product ingestion. Additionally, the analytical strategy allowed us to examine changes in performance of sustained attention block by block for each condition. Despite this, our study is not without limitations. A first limitation is that we did not assess the immediate impact of carbonation on subjective feelings of energy and fatigue. Given that mixed evidence is available on the effects of combining caffeine with refreshing compounds [2], future studies should explore its shorter-term impact on mental and physical energy, to examine the potentially quick effects on mood. A second limitation arises from the need to examine the long-term effects (i.e., >60 min) of caffeinated beverages on mood and cognition [12]. Evidence on the longer-term impact of the combination of caffeine with carbonation is also needed, given that previous studies have reported possible negative effects of different vehicles of caffeine on mood and cognition at 90- and 150-min post-consumption [12]. Such evidence is also needed considering the potential adverse reactions of caffeine consumption, such as anxiety, restlessness and irritability, at high doses (i.e., ≤400 mg/day) [41]. 

## 5. Conclusions

This is the first study to support the contribution of refreshing compounds on the effects of caffeine on sustained attention and feelings of energy and fatigue.

While more research is needed to further understand how carbonation effects interact with caffeine effects, our findings suggest that the combination of CO_2_ with caffeine is likely to display combinatorial effects on time, where CO_2_ generates rapid physiological effects (within 30 min) before caffeine is absorbed and can be effective. Future studies may indeed link these findings to caffeine dosing and timing strategies, to optimize caffeine’s beneficial effects.

## Figures and Tables

**Table 1:** Participant characteristics.

	Mean	Std. Dev.	Median	Minimum	Maximum
**Age (years)**	35.3	8.2	33.0	22.0	48.0
**Height (cm)**	173.2	7.6	174.0	160.0	191.0
**Weight (kg)**	70.8	11.9	73.0	52.0	107.0
**BMI**	23.4	2.5	23.6	19.4	29.3

**Table 2:** Product comparisons for self-reported Physical energy, Physical fatigue, Mental energy, Mental fatigue.

	Difference	Estimated Difference	95% CI	*p*-Value
***Physical energy***				
Product effect				0.0002
Difference	CCB vs. CB	21.79	[−10.89, 54.46]	0.1855
	CCB vs. control beverage	73.72	[40.28, 107.16]	0.0001
	CB vs. control beverage	51.94	[18.44, 85.43]	0.0032
***Physical fatigue***				
Product effect				0.0027
Difference	CCB vs. CB	−16.69	[−48.77, 15.39]	0.2996
	CCB vs. control beverage	−57.91	[−90.3, −25.53]	0.0008
	CB vs. control beverage	−41.22	[−73.81, −8.64]	0.0144
***Mental energy***				
Product effect				<0.0001
Difference	CCB vs. CB	15.1	[−16.88, 47.08]	0.3458
	CCB vs. control beverage	77.5	[44.93, 110.06]	<0.0001
	CB vs. control beverage	62.39	[30.19, 94.6]	0.0003
***Mental fatigue***				
Product effect				<0.0001
Difference	CCB vs. CB	−3.58	[−29.31, 22.14]	0.78
	CCB vs. control beverage	−62.09	[−87.85, −36.33]	<0.0001
	CB vs. control beverage	−58.51	[−84.06, −32.95]	<0.0001

## Data Availability

Data and/or statistical analyses are available upon reasonable request by qualified scientists.
